# Effect of different organic acid additives on the fermentation quality and bacterial community of paper mulberry (*Broussonetia papyrifera*) silage

**DOI:** 10.3389/fmicb.2022.1038549

**Published:** 2022-11-01

**Authors:** Mengxin Li, Qiang Yu, Jinyi Xu, Hong Sun, Qiming Cheng, Yixiao Xie, Chunmei Wang, Ping Li, Chao Chen, Yulong Zheng

**Affiliations:** ^1^College of Animal Science, Guizhou University, Guiyang, China; ^2^Key Laboratory of Animal Genetics, Breeding & Reproduction in the Plateau Mountainous Region, Ministry of Education, Guizhou University, Guiyang, Guizhou, China

**Keywords:** silage, paper mulberry, organic acid, additives, bacterial community

## Abstract

To investigate the effects of different organic acid additives and their concentrations on the fermentation quality and bacterial community of paper mulberry silage, paper mulberry was left untreated (control) or was treated with ethylenediaminetetraacetic acid (EDTA), propionic acid (PA) or citric acid (CA), the amount of each additive was 2 g.kg^−1^ FM, 5 g.kg^−1^ FM and g.kg^−1^ FM. All groups were ensiled for 3, 7, 15, 30 and 60 days. Compared to the control, adding EDTA reduced protein breakdown, preserved more water-soluble carbohydrates of the silages (WSCs, 24.74 g.kg^−1^ DM), and high concentrations of EDTA inhibited the activity of undesirable microorganisms. Adding PA increased the abundance of *Lactiplantibacillus* and decreased the abundance of *Enterococcus*, and it caused a rapid decrease in the pH of the silage at an early stage (from 6.50 to 5.31) while altering the microbiota, and low concentrations of PA resulted in high LA (66.22 g.kg^−1^ DM) concentration and low PA (9.92 g.kg^−1^ DM) concentration at 60 days of ensiling. Different concentrations of additives altered the microbial community of paper mulberry to different degrees. High concentrations of PA and CA can increase the abundance of *Lactiplantibacillus*. High concentrations of CA resulted in a rapid decrease in silage pH at an early stage and higher WSC concentration. These results suggest that EDTA, PA and CA can be used as additives to improve the quality of paper mulberry silage.

## Introduction

In southwest China, the cold weather in winter and the lack of available natural forage resources often lead to a lack of feed supply for livestock in winter and early spring (Li et al., 2016). With the increasing demand for meat and dairy products related safety restrictions, a secure feed supply is the crucial basis for livestock husbandry development (Bateman et al., 2020). Preliminary studies have demonstrated that woody plants with high nutritional value, such as mulberry and paper mulberry leaves, have been used as feed resources to alleviate the problem of insufficient feed supply in recent years (Zhang et al., 2019). Paper mulberry (*Broussonetia papyrifera*, PM) is a deciduous perennial plant of the mulberry family that is fast growing and has strong adaptability to the environment and soil (Peng et al., 2015). It is also rich in amino acids, proteins, vitamins, mineral trace elements, and various beneficial phytochemicals (Guo et al., 2021). Because of its high yield and nutritional value, paper mulberry can be used as a high-quality protein feed for ruminants. However, it is crucial to effectively store woody plant resources to ensure year-round supply. Ensiling is an effective way to preserve the nutrients of forage, and silage is a traditional and global feed source for ruminants, especially in southwestern China, which experiences heavy rainfall and humidity levels at harvest time (Dunière et al., 2013; Du et al., 2021).

Ensiling has been considered as a microbial-driven process (Li et al., 2019), which is based on lactic acid bacteria (LAB) converting water-soluble carbohydrates (WSCs) into organic acids under anaerobic conditions and then decreasing the pH of the silage (Brusetti et al., 2006; Wang et al., 2006). However, due to the low WSC concentration and high buffering capacity of fresh paper mulberry, there is not enough substrate for LAB to ferment and it is thus unable to rapidly reduce the silage pH in the initial stage (Smith, 1962; Cheng et al., 2021). Therefore, it is generally difficult to make high-quality silage from paper mulberry. Fresh paper mulberry contains anti-nutritional factors such as tannins, which can combine with enzymes, sugars, proteins and metal ions in animal rations to produce precipitation, resulting in lower digestibility and absorption of nutrients, which in turn reduces the nutritional value of the ration (Cheng et al., 2021; Guo et al., 2021). Previous research has shown that organic acids can be widely used as silage fermentation inhibitors, which inhibit enzymatic and microbial activity during the ensiling process (In et al., 2013). For example, the addition of metalloprotease inhibitors such as EDTA to forage silage can effectively reduce the degradation of nonprotein nitrogen and protein in the silage and improve its fermentation quality (Kleinschmit et al., 2005). Alternatively, citric acid is safer and cheaper than formic acid and acetic acid as an acidifier and antioxidant (Muck et al., 2018). In addition, lactic acid bacteria can metabolize CA into substances such as diacetyl and acetic acid with flavour, which could improve the palatability of silage (Minervini et al., 2010; Passerini et al., 2013). Moreover, it has been demonstrated that PA is a chemical additive with antifungal properties that also provides a low pH environment at the beginning of fermentation (Fu and Diao, 2007). Organic acids can regulate the change in microbial community structure during silage fermentation, effectively inhibiting the growth and reproduction of yeast, mould and other undesirable microorganisms, improving the aerobic stability of silage, and reducing nutrient loss in feed (Yuan et al., 2017). However, there are few studies on these organic acid additives and their addition concentrations to whole-plant paper mulberry silage. The investigation of the effect of these additives on paper mulberry silage is beneficial to the conservation and utilization of paper mulberry as a fodder resource.

The purpose of our research was to ascertain the effects of organic acid additives (EDTA, PA and CA) and concentrations on the fermentation quality and bacterial communities of paper mulberry silage. Then, the optimum addition concentration and optimum additives were analysed to provide a theoretical basis for the better application of paper mulberry in feed production.

## Materials and methods

### Silage preparation

In 2021, paper mulberry was used as an ensiling material by Guizhou Qianchang Shenghe Modern Agriculture Co., Ltd., located in Changshun, Qiannan Buyi Miao Autonomous Prefecture, Guizhou Province, China (26°1′N, 106°27′E, 930 m above sea level). Paper mulberry was harvested at a height of approximately 100 cm on October 23, which stubble height was approximately 20–30-cm-long per plant. The fresh whole plant was wilted to approximately 70% water concentration and then cut into 2–3-cm length. The chopped materials were randomly divided into 10 piles per group for the following treatments:

- no additive (control; CK).
- treatment (TRE).

The additives selected are EDTA (Jinshan Chemical Test Co., Ltd., Chengdu, China), PA (Shanghai Macklin Biochemical Co., Ltd., Shanghai, China) and CA (Kemiou Chemical Reagent Co., Ltd., Tianjin, China). The different additives were mixed with the chopped forage separately according to Table 1. Each bag was filled with 200 g of fresh forage and then vacuumed and sealed with a sealing machine. Sealed bales of silage were stored at ambient temperature for 3, 7, 15, 30 and 60 days. There were 15 replicate bales per treatment. Three of the bales were randomly selected for sampling at each storage period.

**Table 1:** Concentration of different additives added to paper mulberry silage.

Additive	Concentration (g.kg^−1^ FM)		
	0.2 (%FM)	0.5 (%FM)	0.8 (%FM)
EDTA	2	5	8
CA	2	5	8
PA	2	5	8

EDTA, ethylenediaminetetraacetic acid; CA, citric acid; PA, propionic acid; FM, fresh matter.

### Chemical composition analysis

Ten grams of each fresh silage sample was mixed with 90 ml of sterile water, well blended in a laboratory juicer for 1 min, and then filtered through four layers of cheesecloth. Then, the silage pH and ammonia-N (A-N) and organic acid concentrations were determined in the filtrate sample. The pH of the filtrate was measured using a pH metre (PHS-3E, Shanghai INESA Scientific Instruments Co., Ltd., Shanghai, China). Ammonia-N was determined by the method of Broderick and Kang (1980). Approximately 10 ml of filtrate was subjected to centrifugation (4,500 × g, 15 min, 4°C), and 5.0-μL samples of the supernatant were analysed for the concentration of organic acids, including lactic acid (LA), acetic acid (AA), PA and butyric acid (BA) using high-performance liquid chromatography (HPLC, KC-811 column, Shodex; Shimadzu Co., Ltd., Tokyo, Japan) and the method described by Zhang et al. (2016).

The dry matter (DM) concentration of 150-g samples was analysed after drying at 65°C for 48 h to constant weight. The dried samples were crushed with a mill (DFY-300C, Linda Machinery Co., Ltd., Wenling, China) and then passed through 0.40 mm-mesh sieves for analysis of chemical components. The crude protein (CP) concentration was determined using a kieldahl apparatus (Kjeltec 8,400, FOSS, Sweden) by the method of AOAC (1990). Both neutral detergent fibre (NDF) and acid detergent fibre (ADF) were determined using an Ankom 2000 fibre analyser (Ankom Technology, Fairport, NY) by the method of Van Soest et al. (1991) and expressed on a DM basis. The concentration of AN was analysed using the method described by Broderick and Kang (1980). The WSC concentration was determined by the anthrone method of McDonald et al. (1991).

### Microbial population analysis

The filtered liquor was thoroughly shaken and diluted in a gradient from 10^−3^ to 10^−7^. Selected dilutions were plated on MRS agar medium, Bengal red medium and Eosin-methylene blue medium with concentration gradients of 10^−3^, 10^−5^ and 10^−7^, respectively. The Petri dishes were sealed with sealing film, and those containing MRS agar medium were incubated at 37°C under anaerobic conditions for 48 h, and the number of LAB was counted. The Petri dishes containing Bengal red medium and eosin-methylene blue medium were incubated for 48 h at 37°C in a constant temperature incubator and counted for yeast and *E. coli*. Colonies on each medium were counted separately.

### Bacterial community analysis

The genomic DNA of the samples was extracted by the CTAB method. An appropriate amount of sample was placed in a centrifuge tube and diluted with sterile water to 1 ng/μl. The full-length 16S ribosomal RNA (rRNA) gene was amplified using specific primers (341F: 5′-CCTAYGGGRBGCASCAG-3′; 806R: 5′-GGACTACNNGGGTATCTAAT-3′) with barcodes. Polymerase chain reaction (PCR) was performed using specific primers with Barcode from New England Biolabs’ Phusion^®^ High-Fidelity PCR Master Mix with GC Buffer, and high-performance high-fidelity enzymes to ensure amplification efficiency and accuracy. PCR products were mixed in equal density ratios and purified with a QIAquick^®^ Gel Extraction Kit (QIAGEN). Libraries were constructed using a TruSeq^®^ DNA PCR-Free Sample Preparation Kit according to the manufacturer’s recommendations. The constructed library was quantified by Qubit and quantitative PCR (Q-PCR), and the libraries were sequenced using NovaSeq6000. Raw sequences were processed by Novogene Bio Technology Co., Ltd., Beijing, China, for annotating the taxonomic information in the SSUrRNA Database of Silva Database. After phylogenetic relationships were constructed, alpha diversity was analysed on the Magic platform.^1^

### Statistical analysis

Data on the silages on the 3rd, 7th, 15th, 30th and 60th days of storage were subjected to two-way ANOVA for a 5 (days of storage) × 3 (treatments) factorial arrangement. Data on changes in chemical composition, microbial population and bacterial community indices during storage were compared using Duncan’s test in the SPSS 26.0 programme (SPSS Inc., Chicago, IL, United States). Differences were considered statistically significant only when the probability level was lower than 0.05 (*p <* 0.05). In addition, the interactions between some silage parameters and bacterial community/function prediction of the bacterial community of paper mulberry silages were presented by establishing their Spearman correlations which were compared using OriginPro 2021 program and visualized in heatmaps. All figures were generated using OriginPro 2021.

## Results and discussion

### Chemical composition of fresh paper mulberry

The chemical composition of paper mulberry before ensiling is presented in Table 2. The DM concentration of fresh paper mulberry was approximately 369.43 g.kg^−1^ FM (fresh matter, FM), which was similar to that found by Zhang et al. (2019). A previous study (Cheng et al., 2021) found that the concentrations of NDF and ADF in paper mulberry were approximately 303.92 g.kg^−1^ DM and 200.83 g.kg^−1^ DM, respectively, which are significantly lower than our findings. We found that the NDF concentration of paper mulberry was approximately 677.20 g.kg^−1^ DM, and the ADF concentration was approximately 310.80 g.kg^−1^ DM. Similar to the findings of Cheng et al. (2021), fresh paper mulberry had a WSC concentration of approximately 75.83 ± 0.63 g.kg^−1^ DM. However, the CP concentration was approximately 99.43 ± 0.25 g.kg^−1^ DM, which was different from the results of Sun et al. (2022), who reported that the CP concentration of fresh paper mulberry was approximately 164.71 g.kg^−1^ DM. The reason for the low concentration of CP but high concentration of NDF and ADF in our study may be that previous studies focused on the leaves of paper mulberry, while our study focused on the whole paper mulberry plant. Another reason may be that the harvest time was autumn-winter, and the nutrient concentration of plants was low when the last crop was harvested. The WSC concentration (>55 g.kg^−1^ DM) of fresh paper mulberry was sufficient for the growth of yeasts at the early stage of ensiling and of LAB at the subsequent stage (Li et al., 2019). The WSC concentration of fresh paper mulberry in our study (approximately 75 g.kg^−1^ DM) was sufficient as a substrate for the propagation and growth of yeasts in the initial stage of ensiling and of LAB in the successive stage (Li et al., 2016). The LAB concentration of fresh paper mulberry was about 5.43 log cfu·g^−1^ FM, which was similar to Cheng et al. (2021), while that of yeasts was about 6.45 log cfu·g^−1^ FM, which was similar to Zhang et al. (2022).

**Table 2:** Chemical composition of fresh paper mulberry.

Items	Paper mulberry
DM g.kg^−1^ FM	369.43 ± 0.78
CP g.kg^−1^ DM	99.43 ± 0.25
WSC g.kg^−1^ DM	75.83 ± 0.63
NDF g.kg^−1^ DM	677.20 ± 0.66
ADF g.kg^−1^ DM	310.80 ± 0.61
pH	6.50 ± 0.10
LAB log cfu·g^−1^ FM	5.43 ± 0.06
Yeasts log cfu·g^−1^ FM	6.45 ± 0.08

FM, fresh matter; DM, dry matter; CP, crude protein; WSCs, water-soluble carbohydrates; NDF, neutral detergent fibre; ADF, acid detergent fibre; LAB, lactic acid bacteria; cfu, colony-forming units.

### Chemical composition of paper mulberry silage

The influence of different additives and their concentrations on the DM, CP, and WSC concentrations of paper mulberry during ensiling is shown in Table 3, and the influence on NDF and ADF at 60 days is shown in Table 4. In silage fermentation, the moisture concentration of the material is an important indicator due to the requirements of LAB for growth and reproduction (Wang et al., 2018; Xu et al., 2020). In this study, the trend of DM changes was similar between CK and TRE; most of the DM concentration of the TRE groups increased at the beginning of ensiling, with a high DM concentration at 15 days, and then decreased with increasing ensiling time. This is probably because microorganisms need more water to reproduce (Hu et al., 2009). It is worth mentioning that the DM concentration of 0.5P was as high as 410 g.kg^−1^ FM, which indicated that the addition of PA can effectively inhibit the growth of some microorganisms, thus minimizing the loss of silage DM (Gheller et al., 2021). At 60 days of ensiling, the DM of the TRE group was higher than that of CK, possibly because the organic acid additives inhibited the growth and reproduction of undesirable microorganisms to preserve the nutrition of the forage. After 60 days of ensiling, we observed that additives had a significant effect on the NDF concentration (*p* < 0.05) but not on the ADF concentration of paper mulberry. It might be that ADF is mainly composed of cellulose and lignin, which is not easy to degrade, while NDF contains more degradable components, and acid hydrolysis caused by the relatively low pH and consumption by microorganism could result in lower concentration of NDF (Phillip et al., 1990). Compared with that of CK, the NDF concentration of paper mulberry silage was significantly reduced and the degradation rate was increased at different concentrations of EDTA, and the low concentration of EDTA was more effective than the high concentration. Further research is needed to explain this phenomenon.

**Table 3:** Chemical characterization of paper mulberry ensiled with different treatment during fermentation.

Item	Ensiling days (D)	Treatment (T)										SEM	*p* value		
		0.2E	0.5E	0.8E	0.2P	0.5P	0.8P	0.2C	0.5C	0.8C	CK		D	T	D × T
DM (g.kg^−1^ FM)	3	372.62Bbcd	371.93Bcd	379.26Babc	371.01Bcd	386.07Ba	381.95Bab	365.61 Bd	392.77Bab	374.24Bcd	376.91Ba	0.09	^***^	^***^	0.190
	7	367.70Cbcd	363.11Ccd	368.14Cabc	359.77Ccd	368.03Ca	377.20Cab	364.53Cd	386.79Cab	370.21Ccd	382.37Ca				
	15	383.92Abcd	377.94Acd	388.44Aabc	379.60Acd	410.14Aa	391.31Aab	368.78Ad	382.41Aab	374.38Acd	397.46Aa				
	30	369.67BCbcd	373.90BCcd	373.69BCabc	367.64BCcd	371.64BCa	378.69BCab	361.15BCd	369.43BCab	362.77BCcd	393.52BCa				
	60	373.35BCbcd	373.26BCcd	381.30BCabc	379.09BCcd	378.77BCa	380.78BCab	369.80BCd	375.95BCab	367.55BCcd	364.64BCa				
CP (g.kg^−1^ DM)	3	93.82Bcd	100.68Bab	101.79Ba	82.71Bfg	85.68Bg	92.87Be	92.41Be	100.72 Bd	86.35Bef	94.17Bbc	0.04	0.12	^***^	0.278
	7	100.72Acd	102.40Aab	108.38Aa	88.18Afg	84.39Ag	88.83Ae	94.31Ae	94.15Ad	90.42Aef	106.71Abc				
	15	100.80ABcd	103.09ABab	104.11ABa	85.37ABfg	86.22ABg	89.61ABe	89.18ABe	96.32ABd	89.62ABef	104.42ABbc				
	30	102.40ABcd	102.34ABab	106.62ABa	89.85ABfg	83.23ABg	92.27ABe	92.79ABe	94.56ABd	90.28ABef	97.35ABbc				
	60	93.62ABcd	106.62ABab	104.56ABa	84.07ABfg	86.05ABg	92.15ABe	90.14ABe	92.44ABd	88.43ABef	97.24ABbc				
A-N (g.kg^−1^ DM)	3	4.88Ea	4.15Ecd	3.60Ed	5.93Eb	5.96Ebc	5.25Ebc	5.33Eb	5.79Ebc	5.18Ebcd	4.43Eb	0.04	^***^	^***^	0.198
	7	12.65 Da	6.85Dcd	5.92Dd	9.93Db	10.09Dbc	10.92Dbc	12.05Db	12.31Dbc	9.44Dbcd	11.54Db				
	15	35.73Ba	21.29Bcd	18.15 Bd	28.28Bb	27.20Bbc	23.59Bbc	26.32Bb	19.51Bbc	17.28Bbcd	27.71Bb				
	30	24.75Ca	16.04Ccd	16.77Cd	20.80Cb	19.48Cbc	20.37Cbc	20.03Cb	19.81Cbc	21.00Cbcd	20.87Cb				
	60	44.07Aa	22.54Acd	18.44Ad	35.11Ab	22.85Abc	28.95Abc	35.25Ab	29.00Abc	27.78Abcd	27.92Ab				
WSC (g.kg^−1^ DM)	3	60.41Abc	55.61Aab	52.37Aab	64.28Aab	47.78Abc	67.10Aa	53.02Ac	57.70Abc	61.06Abc	52.26Ad	0.10	^***^	^***^	0.187
	7	56.71Bbc	57.11Bab	46.86Bab	53.02Bab	42.65Bbc	50.30Ba	39.09Bc	38.88Bbc	38.95Bbc	18.52 Bd				
	15	52.58Abc	67.38Aab	69.77Aab	66.66Aab	52.42Abc	73.52Aa	44.83Ac	57.14Abc	47.60Abc	21.27Ad				
	30	24.73Bbc	45.53Bab	49.86Bab	44.31Bab	61.07Bbc	58.39Ba	31.67Bc	39.17Bbc	55.77Bbc	19.74 Bd				
	60	24.03Cbc	43.18Cab	41.21Cab	38.99Cab	37.74Cbc	55.47Ca	35.12Cc	29.28Cbc	39.40Cbc	18.44Cd				

CK, control; E, EDTA; P, propionic acid; C, citric acid; 0.2, concentration of additive is 2 g.kg^−1^ FM; 0.5, concentration of additive is 5 g.kg^−1^ FM; 0.8, concentration of additive is 8 g.kg^−1^ FM; DM, dry matter; CP, crude protein; A-N, ammonia nitrogen; WSC, water-soluble carbohydrate. Different uppercase letters indicate significant differences among different ensiling days within the same treatment (*P* < 0.05); different lowercase letters indicate significant differences among different treatments within the same ensiling day (*p* < 0.05); SEM = standard error of the mean; D = ensiling day, T = treatment, D × T = interaction between ensiling day and treatment.

*** represents *p* < 0.001.

**Table 4:** Chemical composition of paper mulberry silage at 60 days.

Treatment	NDF (g.kg^−1^ DM)	ADF (g.kg^−1^ DM)
CK	722.31 ± 12.15a	363.7 ± 8.21
0.2E	640.33 ± 12.06 cd	356.66 ± 8.27
0.5E	630.38 ± 20.3d	357.51 ± 22.72
0.8E	665.11 ± 20.61bcd	354.93 ± 5.11
0.2P	684.7 ± 28.32abc	377.43 ± 12.55
0.5P	714.96 ± 4.92ab	383.85 ± 9.68
0.8P	682.75 ± 20.91abcd	362.74 ± 17.5
0.2C	694.22 ± 17.52abc	373.19 ± 22.18
0.5C	690.14 ± 7.51abc	336.29 ± 41.94
0.8C	708.23 ± 4.73ab	339.41 ± 12.8
SEM	6.91	5.69
*p* -value	0.012	0.753

NDF, neutral detergent fibre; ADF, acid detergent fibre; CK, control; E, EDTA; P, propionic acid; C, citric acid; 0.2, concentration of additive is 2 g.kg^−1^ FM; 0.5, concentration of additive is 5 g.kg^−1^ FM; 0.8, concentration of additive is 8 g.kg^−1^ FM; Different lowercase letters indicate significant differences among treatments within the same ensiling day (*p* < 0.05); SEM, standard error of the mean. DM, Dry matter.

The WSC concentration of silage plays a crucial role in LA production (Li et al., 2022). Throughout ensiling, the WSC concentration of all TRE groups was higher than that of the CK group, which indicated that all organic acid additives could reduce the decrease in WSC. At the same time, we observed that the WSC concentration of the CK group decreased to 18.52 g.kg^−1^ DM on the 7th day, which may be due to the increasing consumption of WSC caused by the propagation of undesirable microorganisms in the early stage of ensiling (Li et al., 2022). Similar to our findings, a previous study (Li et al., 2021) demonstrated that inoculation with additives could retain more WSC in mulberry silage. We also discovered that the WSC concentration increased with increasing EDTA concentration, possibly because the high concentration of EDTA inhibited the activity of harmful microorganisms, decreased the utilization of WSC. Moreover, the WSC provided more fermentation substrates for LAB, which in turn produced more LA and decreased the pH of the silage. Compared with CK, the use of EDTA and CA preserved more CP concentration in the paper mulberry silage than the use of PA, especially EDTA. Rooke (1985) found that plant proteases played a major role in the proteolysis of alfalfa during ensiling. Ethylenediaminetetraacetic acid is a protease inhibitor that inhibits plant protease activity, which can directly reduce proteolysis and/or the formation of protein-polyphenol complexes in tannin-containing silage (Lee et al., 2008). Similar results were obtained by Guo et al. (2011), who suggested that the addition of a metallopeptidase inhibitor to ryegrass silage could inhibit the degree of protein degradation in the silage.

As an antioxidant, citric acid can inhibit the activity of proteolytic enzymes. In et al. (2013) found that CA can inhibit the reproductive activities of undesirable microorganisms such as mould and yeast. Compared with CK, the concentration of A-N in the 0.5E and 0.8E treatment was lower, probably because of the increase in EDTA concentration, which decreased the activity of undesirable microorganisms, and less A-N was generated, resulting in a higher CP concentration being preserved in the silage.

### Fermentation profile and microbial population paper mulberry silages

**Figure 1:**
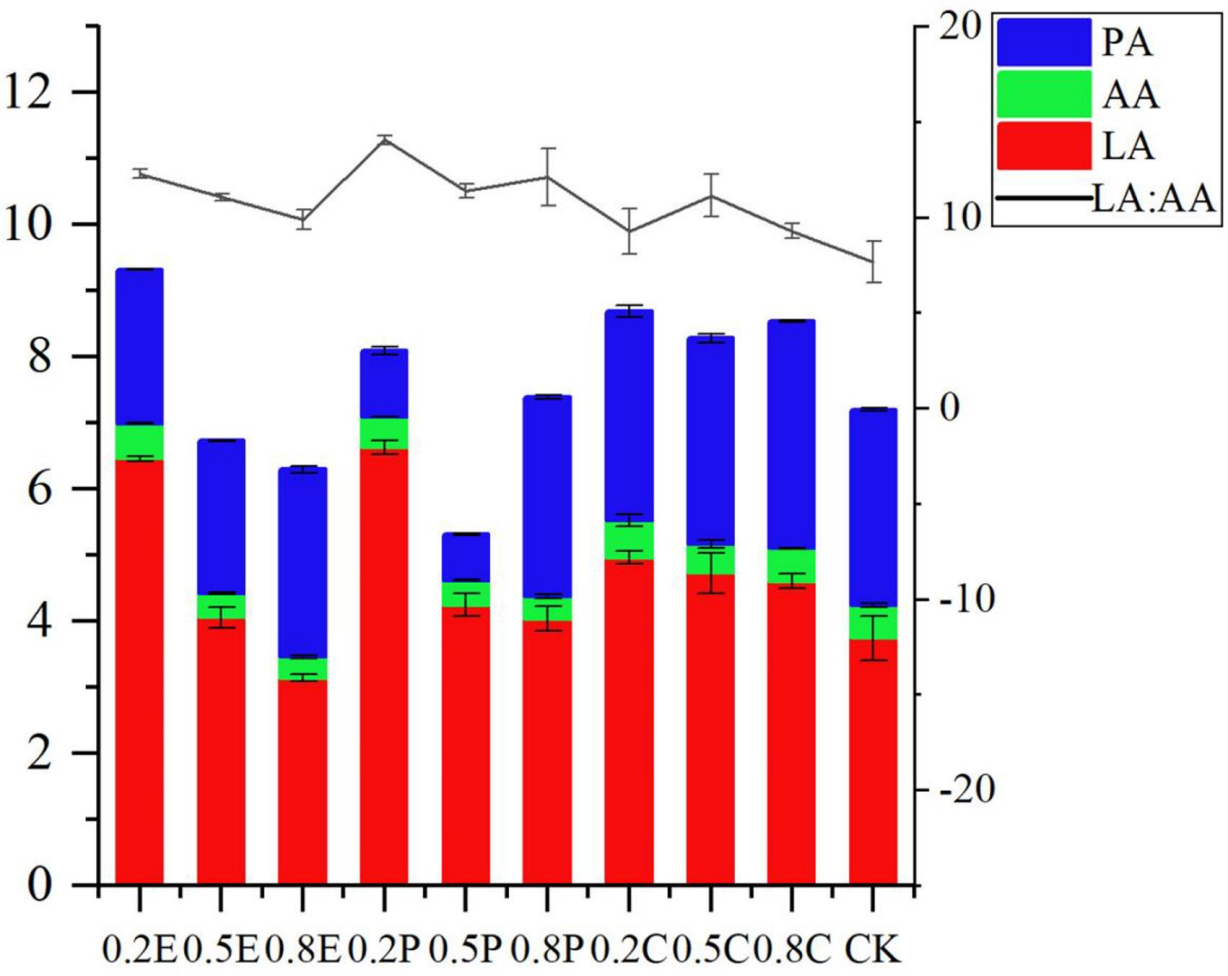
Organic acid concentration of paper mulberry silage at 60 days. CK, control; E, EDTA; P, propionic acid; C, citric acid; 0.2, concentration of additive is 2 g.kg^−1^ FM; 0.5, concentration of additive is 5 g.kg^−1^ FM; 0.8, concentration of additive is 8 g.kg^−1^ FM; LA, lactic acid; AA, acetic acid; PA, propionic acid; LA: AA, lactate/acetic acid ratio.

Table 5 and Figure 1 show the fermentation quality of paper mulberry silage. In our study, the interaction of D × T existed for pH (*p* < 0.001). The pH of the additive-treated silage decreased during the first 7 days and then remained stable or increased during ensiling. Lv et al. (2020) found that the pH of CA-treated silage increased with the extension of the storage period, which was similar to our results. The increase in pH during fermentation processes may result in the production of acetic and/or butyric acid by the consumption of LA during long periods of fermentation (Blanco et al., 2019). The additives selected in our study were all organic acids, which can promote an acidic environment and facilitate the growth of LAB during silage storage (Gheller et al., 2021). However, the positive effect decreases with the extension of the storage period. Due to the strong buffering capacity of paper mulberry (Cheng et al., 2021), although the addition of propionic acid declined the silage pH in the early stage, the decline rate was inhibited after 3 days of ensiling. Therefore, the rate of pH decline was suppressed in the treatment group with PA after 3 days of ensiling. During the first 3 days, the pH of silage in the 0.8C treatment decreased the most, probably due to the high concentration of CA addition. CA, as an acidifier and carbon source, can rapidly reduce the pH and increase carbohydrate concentration in the early stage of ensiling, which is consistent with the study of Ke et al. (2017).

**Table 5:** Fermentative profile of paper mulberry ensiled with different treatment during fermentation.

Items	Ensiling days (D)	Treatment (T)										SEM	p value		
		0.2E	0.5E	0.8E	0.2P	0.5P	0.8P	0.2C	0.5C	0.8C	CK		D	T	D × T
pH	3	6.15Aa	6.04Aa	5.53Aabc	5.31Bbc	5.79Bab	5.92ABab	6.03Aa	6.09Aa	5.11ABc	5.86Aab	0.025	^***^	^***^	^***^
	7	5.08Bcd	5.43Babc	5.24Abcd	5.37Babc	5.58Bab	5.75ABa	4.84Bde	4.88Bde	5.12ABcd	4.63Be				
	15	5.97Aab	5.57Bbc	5.22Acd	6.44Aa	6.26Aa	6.24Aab	5.92Aab	5.17Bcd	4.78 Bd	4.71BCd				
	30	5.10Bde	5.77ABbc	5.25Acde	6.19Aab	6.51Aa	5.52Bcd	5.29ABcde	4.74Be	5.57Abcd	4.67BCe				
	60	4.66Bcd	4.93Cc	4.61Bcd	5.97Aa	5.45Bb	5.36Bb	5.48ABb	4.45 Bd	4.89Bcd	4.95Bcd				
LAB (log cfu·g^−1^ FM)	3	5.48Dd	5.69CDd	6.49Ab	6.93Aa	6.59Bab	6.09Bc	6.48Ab	6.62Aab	6.93Aa	6.32BCbc	0.016	^***^	^***^	^***^
	7	6.99Aa	5.86BCc	5.83Bc	7.01Aa	7.00Aa	6.96Aa	7.00Aa	6.79Ab	6.81Ab	6.99Aa				
	15	6.55Bbc	6.56Abc	6.53Abc	6.62Bbc	6.43BCc	6.38Bc	6.58Abc	6.72Ab	6.46Bbc	7.00Aa				
	30	6.24Cabc	6.00Bc	6.09Bbc	6.42Cab	6.34Cab	6.32Bab	6.51Aa	6.28Babc	6.20Babc	6.43Ba				
	60	6.26Cab	5.57Dd	5.95Bbc	5.47Dd	5.67Dcd	5.57Cd	5.58 Bd	6.53ABa	5.78Ccd	6.13Cb				
Yeasts (log cfu·g^−1^ FM)	3	5.60Cb	5.59b	6.38Aab	6.34ab	5.76Bab	5.54Bb	6.21ab	6.10ab	6.64Aa	5.56b	0.039	^***^	^***^	0.198
	7	6.83Aab	5.09ef	5.00Bf	6.94a	6.96Aa	5.63Bde	6.88ab	6.04 cd	6.35Abc	5.89 cd				
	15	6.26Ba	5.72abc	5.54Bbcd	6.25a	6.01Bab	5.98Aab	5.85abc	5.28 cd	5.34Bcd	5.00d				
	30	ND	ND	ND	ND	ND	ND	ND	ND	ND	ND				
	60	ND	ND	ND	ND	ND	ND	ND	ND	ND	ND				

CK, control; E, EDTA; P, propionic acid; C, citric acid; 0.2, concentration of additive is 2 g.kg^−1^ FM; 0.5, concentration of additive is 5 g.kg^−1^ FM; 0.8, concentration of additive is 8 g.kg^−1^ FM; LAB, lactic acid bacteria; cfu, colony-forming units; FM, fresh matter; Different uppercase letters indicate significant differences among different ensiling days within the same treatment (*P* < 0.05); different lowercase letters indicate significant differences among different treatments within the same ensiling day (*p* < 0.05); SEM = standard error of the mean; ND, not detected; D = ensiling day, T = treatment, D × T = interaction between ensiling day and treatment.

*** represents *p* < 0.001.

On day 60, with the increase in EDTA concentration, the LA concentration and the ratio of lactate/acetate of silage decreased, and the organic acid concentration was lower. The reason may be that EDTA inhibits microbial reproduction and delays LAB fermentation so that the rate of pH reduction is slower than that of CK. Propionic acid is extremely soluble and permeable which might alter the silage microbiota at the beginning of ensiling, resulting in certain more acid-tolerant LAB becoming the dominant bacteria during silage fermentation (Adesogan and Salawu, 2002; Kleinschmit et al., 2005). Therefore, adding low concentration of PA can significantly increase the concentration of LA compared with CK. Interestingly, the concentration of PA in the 0.2P and 0.5P groups was significantly lower than that in other groups, but only the 0.2P treatment had a significantly higher LA concentration than the other treatments. In general, homogeneous fermentation is the main type of silage fermentation in temperate and cool regions (Zhou et al., 2016; Chen et al., 2020a), while a low ratio of lactate/acetate (<2.5 ~ 3.0) is generally regarded as the main fermentation type for tropical silage (Guan et al., 2018; Wang et al., 2019). In our study, the ratio of lactate/acetate was higher than 3.0 in all groups, which means that all groups underwent homogeneous fermentation. All ratios of lactate/acetate in the TRE groups were higher than those in the CK group; in addition, the ratio of lactate/acetate at 0.2P was the highest, which indicated the dominance of *Lactobacillus* fermentation in this treatment. A study by Pahlow et al. (2003) demonstrated that LA produced in silage can be converted to AA and PA under anaerobic conditions. Therefore, the amount of organic acids and PA in CA-treated silage was higher, but the concentration of LA was low. At 60 days, butyrate acid was not detected in any of the silages, consistent with the findings of Cheng et al. (2021).

The dynamics of the LAB and yeasts populations of paper mulberry silages are listed in Table 5. The number of LAB increased at the initial stage and then decreased, as many LAB strains are relatively intolerant to lower pH values (Ohmomo et al., 2002). In our study, the interaction of D × T had a strikingly significant (*p* < 0.001) impact on the LAB population in paper mulberry silage. Before 15 days of fermentation, additives resulted in lower numbers of yeasts because of the decrease in pH. Yeasts were not detected in any of the samples after 30 days, which was due to the anaerobic environment during the late period of silage fermentation (Zhang et al., 2021), indicating better fermentation performance.

### Bacterial community diversity and abundance of paper mulberry silage

**Figure 2:**
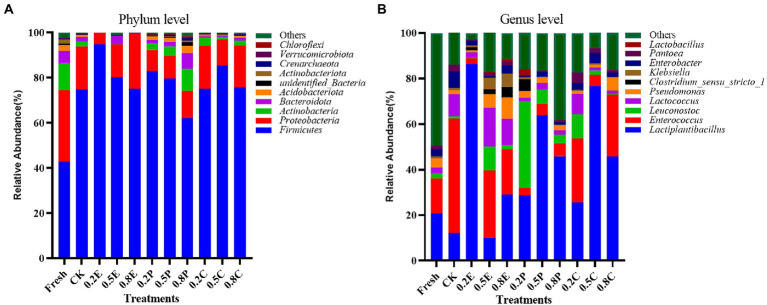
Relative abundance of bacteria at day 60 identified at **(A)**, the phylum level and **(B)**, the genus level according to the classification of the microbial community of paper mulberry silage. CK, control; E, EDTA; P, propionic acid; C, citric acid; 0.2, concentration of additive is 2 g.kg^−1^ FM; 0.5, concentration of additive is 5 g.kg^−1^ FM; 0.8, concentration of additive is 8 g.kg^−1^ FM.

The bacterial alpha diversity of silage, including observed species, Good’s coverage, ACE, Chao1 estimation, and Shannon index of bacteria, is shown in Table 6. Alpha diversity reflects the sequencing depth index (observed species and Good’s coverage), bacterial abundance (ACE and Chao1) and species diversity (Shannon index) in the samples (Du et al., 2021). The Good’s coverage of most samples was above 0.97, which indicated that most bacteria could be detected by high-throughput sequencing technology, making it feasible to analyse microbial communities. During storage, fewer species were observed in the silage than in fresh paper mulberry. Under anaerobic conditions, microorganisms on plants were mostly replaced by LAB populations during ensiling, with a sharp decrease in observed species (Chen et al., 2020a). Therefore, after 60 days of ensiling, the alpha diversity decreased in all silages. As expected, 0.2E-treated silage had lower observed species, ACE, Chao1 estimates and Shannon index values than CK silages, which may be due to the higher relative abundance of *Lactiplantibacillus* (Figure 2), which could inhibit most of the microbial growth. A previous study indicated that lower diversity in microbial communities of silages is usually caused by an increase in the abundance of dominant bacteria (Li et al., 2022). However, we found that the observed species, ACE, Chao1 estimator and Shannon index in 0.8P- and 0.2C-treated silage were higher than those in CK silage, which may contribute to the higher pH, resulting in the increasing proportion of undesirable microorganisms and heterotypic fermentation LAB and leading to higher microbial diversity and abundance (Hu et al., 2009). Moreover, Ren et al. (2019) also reported that the bacterial alpha diversity index of sugarcane top silage increased with longer storage periods. In addition, bacterial alpha diversity may increase due to higher pH during complete and/or incomplete silage fermentation (Chen et al., 2020b).

**Table 6:** Diversity and richness of bacterial microbiota of paper mulberry ensiled with different treatments during fermentation.

Treatment	Observed species	Good’s coverage	ACE	Chao1	Shannon
Fresh	146 ± 6	0.985 ± 0.004	155.36 ± 9.36	149.18 ± 7.43	5.36 ± 0.18
CK	64 ± 10	0.986 ± 0.002	75.06 ± 10.69	69.15 ± 11.29	2.99 ± 0.33
0.2E	25 ± 4	0.997 ± 0.001	27.49 ± 4.45	25.70 ± 4.18	1.03 ± 0.34
0.5E	42 ± 10	0.995 ± 0.001	45.39 ± 8.85	42.73 ± 9.63	2.90 ± 0.95
0.8E	39 ± 1	0.996 ± 0.001	41.66 ± 1.59	39.85 ± 1.31	3.28 ± 0.27
0.2P	68 ± 18	0.987 ± 0.005	77.10 ± 22.57	72.03 ± 20.28	2.80 ± 0.49
0.5P	71 ± 26	0.985 ± 0.005	82.91 ± 29.49	76.66 ± 27.42	2.45 ± 1.13
0.8P	123 ± 28	0.975 ± 0.004	143.75 ± 29.54	133.19 ± 28.07	4.06 ± 1.11
0.2C	68 ± 17	0.986 ± 0.002	78.78 ± 17.63	72.86 ± 17.18	3.26 ± 0.70
0.5C	40 ± 6	0.990 ± 0.001	49.23 ± 7.69	43.51 ± 6.52	1.56 ± 0.57
0.8C	58 ± 16	0.988 ± 0.002	69.27 ± 15.36	62.50 ± 15.79	2.24 ± 0.67
SEM	4.67	0.001	5.11	4.84	0.208
*p*-value	^***^	^**^	^***^	^***^	^*^

CK, control; E, EDTA; P, propionic acid; C, citric acid; 0.2, concentration of additive is 2 g.kg^−1^ FM; 0.5, concentration of additive is 5 g.kg^−1^ FM; 0.8, concentration of additive is 8 g.kg^−1^ FM; SEM, standard error of means. Significant differences (*P* < 0.05) among the different treatments are indicated by lowercase letters.

*** represents *p* < 0.001;

** represents *p* < 0.01;

* represents *p* < 0.05.

**Figure 3:**
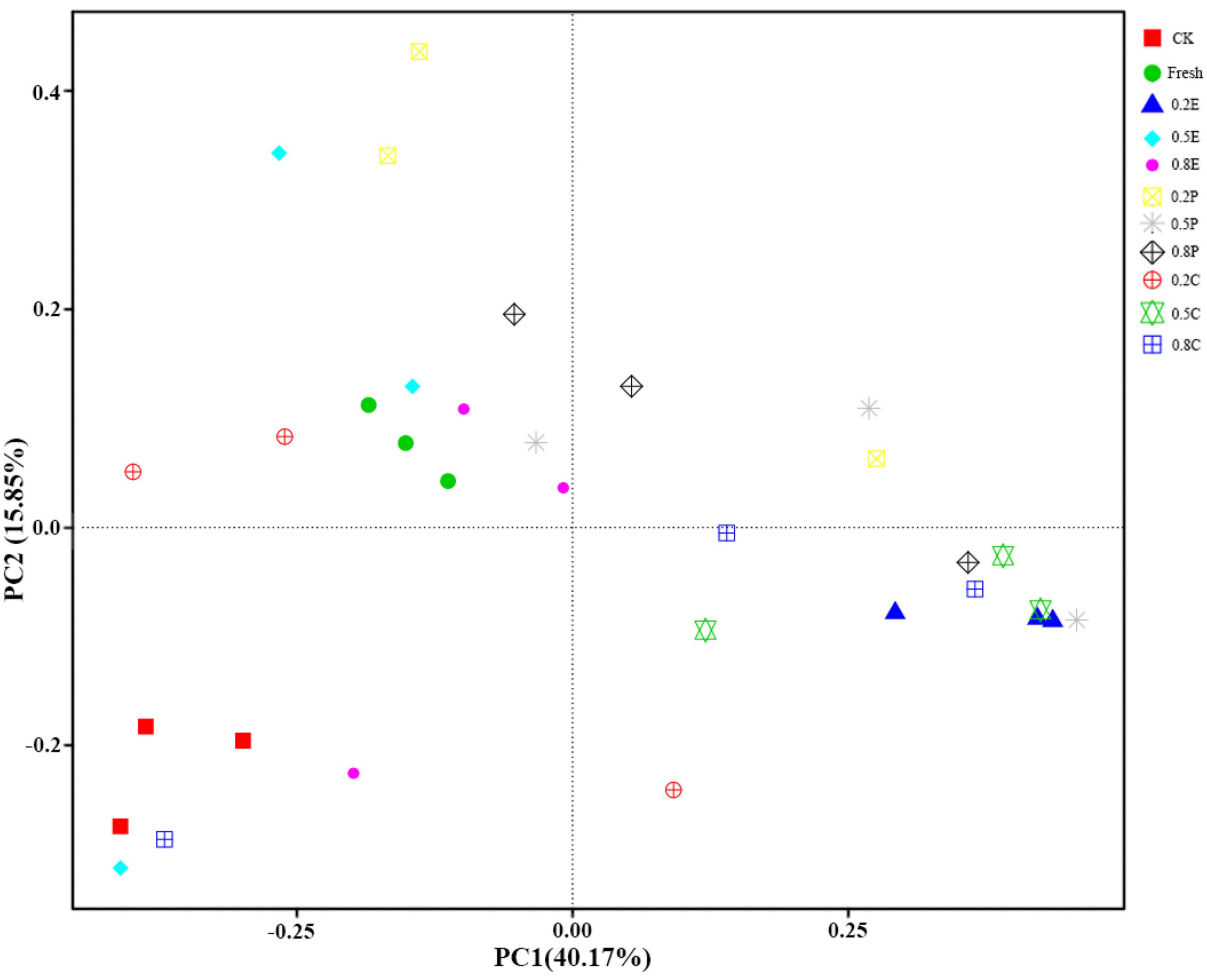
Principal coordinates analysis (PCoA) of bacterial communities of paper mulberry silage on day 60. CK, control; E, EDTA; P, propionic acid; C, citric acid; 0.2, concentration of additive is 2 g.kg^−1^ FM; 0.5, concentration of additive is 5 g.kg^−1^ FM; 0.8, concentration of additive is 8 g.kg^−1^ FM.

Principal coordinate analysis (PCoA) was used to assess the difference in bacterial communities among the silage samples. As shown in Figure 3, the two axes together accounted for 56.02% of the total variance, and principal component 1 (PC1) and principal component 2 (PC2) accounted for 40.17 and 15.85% of the variance, respectively. The fresh material was in the second quadrant, while CK was in the third quadrant, which indicated that there was a significant difference in microbial communities between fresh paper mulberry and paper mulberry silage after 60 days of silage. Most of the treatments were distributed in the second and fourth quadrants, which were significantly different from CK, which means that different treatment groups had large and different impacts on the microbial community of the paper mulberry silage. This is also reflected by the relative abundance of bacteria in Figure 2. This result is consistent with previous findings that microorganisms in silage are slowly replaced by LAB under anaerobic conditions (Ni et al., 2017).

In Figure 2, the bacterial community composition of the samples is shown as stacked columns at the phylum and genus levels. Figure 2A shows that the paper mulberry silage was dominated by Firmicutes and Proteobacteria after 60 days of ensiling, which is consistent with previous studies (Du et al., 2021). Firmicutes are important for the production of organic acids such as LA and AA in the late fermentation stage. Compared with the fresh groups, the relative abundance of Firmicutes increased significantly after 60 days of ensiling and became the dominant phylum in all silage samples. The relative abundance of Firmicutes in the 0.2E-treated silage was the highest. At the genus level (Figure 2B), the main genera in the fresh and CK treatments were *Enterococcus* (15.33 and 50.43%, respectively), *Lactiplantibacillus* (20.72 and 12.09%, respectively) and Others (13.913 and 49.54%, respectively). In the TRE groups, the relative abundance of *Lactiplantibacillus* in 0.2E was 86.23%, followed by 76.75 and 63.94% in 0.5C and 0.5P, respectively. The addition of the additives increased the relative abundance of *Lactiplantibacillus.* A previous study indicated that *Lactobacillus* and *Lactococcus* are functional bacteria that could be used to improve the quality of silage (Yang et al., 2016). *Lactobacillus* plays a crucial role in LAB fermentation, as it can grow and reproduce rapidly, produce LA by fermenting WSC, reduce the pH value of silage to preserve nutrients, and maintain high viability at low pH (Dunière et al., 2013). In contrast, *Enterococci* are considered unwanted bacteria because they may compete with LAB to utilize fermentation products (Rooke and Hatfield, 2003). In 0.5E, the relative abundance of *Enterococcus* was high (29.53%), and the concentration of LA was low, but a high WSC concentration was preserved (Table 2), which may be because the added amount of EDTA was high and inhibited the fermentation of silage (Guo et al., 2011)*.* Therefore, *Enterococcus* failed to replace *Lactiplantibacillus* and became the dominant bacterial community in the early stage. Some LAB, enterobacteria and *Clostridium* have proteolytic activity (Rooke and Hatfield, 2003). In the low-concentration EDTA groups, such as 0.2E and 0.5E, the enterobacteria and *Clostridium* concentrations were lower than in some other groups, such as 0.2P and CK. It is worth mentioning that we also observed a high abundance of *Lactococcus* (17.07%) in 0.5E. Similar results were obtained by Guo et al. (2011), EDTA treatment reduced the decomposition rate of CP in silage by undesirable microorganisms and effectively preserved CP (Table 2), the reason may be that EDTA belong to the metalloprotease inhibitors and lower pH of silage. However, 0.5E contained a high abundance of *Klebsiella* (5.16%) but a high CP concentration, which was not consistent with the study of Scherer et al. (2015), who reported that *Klebsiella* bacteria could degrade amino acids to produce ammonia and biogenic amines, and the mechanism still needs to be further explored.

### Function prediction and correlation analysis of the bacterial community based On some silage parameters

**Figure 4:**
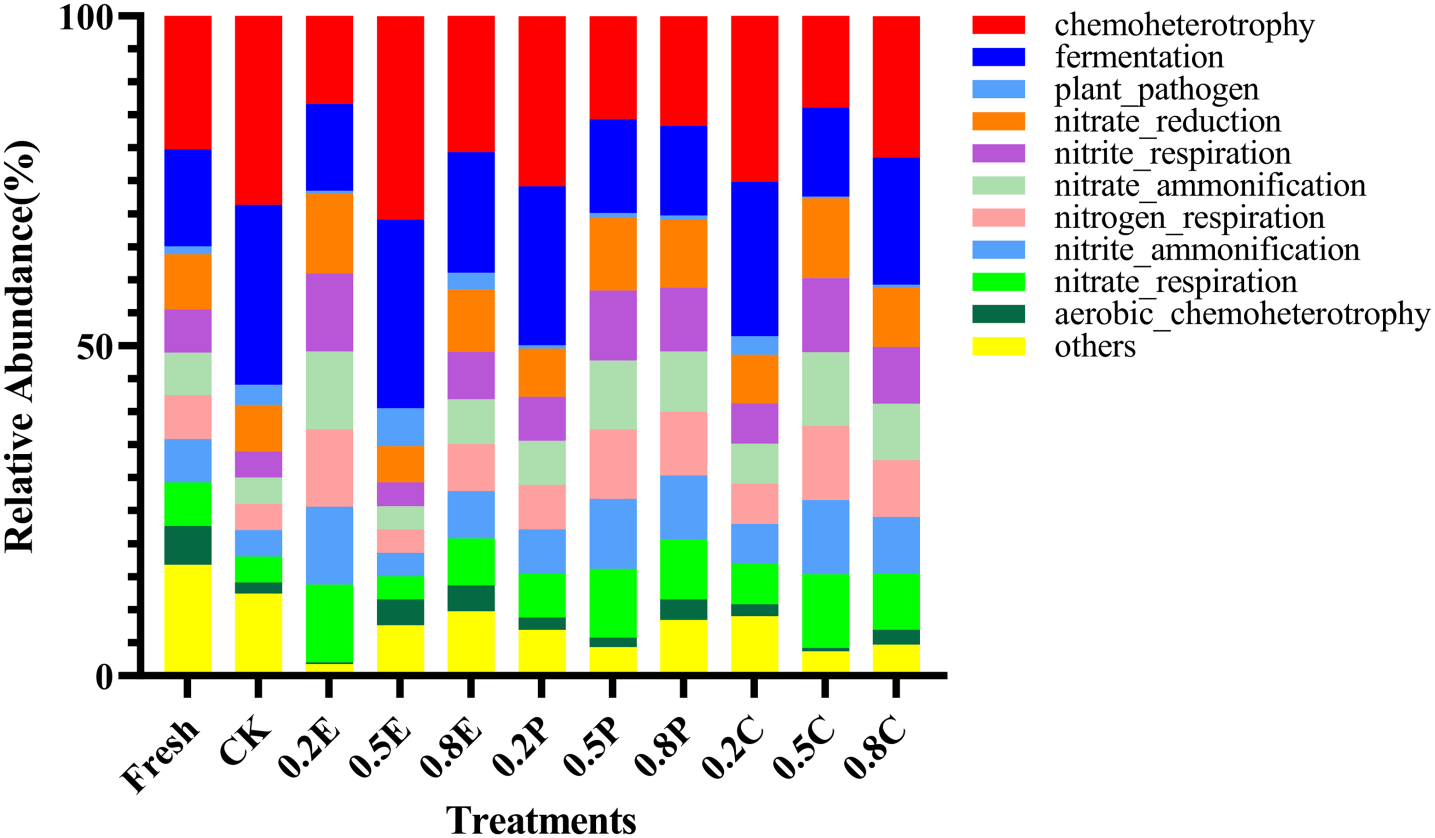
Function prediction of the bacterial community in paper mulberry silage on day 60. CK, control; E, EDTA; P, propionic acid; C, citric acid; 0.2, concentration of additive is 2 g.kg^−1^ FM; 0.5, concentration of additive is 5 g.kg^−1^ FM; 0.8, concentration of additive is 8 g.kg^−1^ FM.

In Figure 4, the functional predictions of the bacterial communities of paper mulberry silage after 60 days of ensiling are shown in the form of stacked bars. Chemoheterotrophy is the primary function of the microbial communities, followed by fermentation, plant pathogen, nitrate reduction, and nitrite respiration. The bacterial community of CK had a higher relative abundance of chemoheterotrophy and protein functions than that of fresh groups, indicating that carbon fixation was inhibited and carbon and energy were obtained by oxidizing organic components for the growth of a large amount of bacteria (Zhang et al., 2019). In addition, for 0.2E, 0.5P, 0.8P, 0.5C, or 0.8C, nitrate reduction, nitrite respiration, nitrate ammonification, nitrogen respiration, nitrite ammonification, and nitrate respiration were higher, which may have caused the lower CP concentration in these silages (Table 3), which is similar to the results of Li et al. (2022).

**Figure 5:**
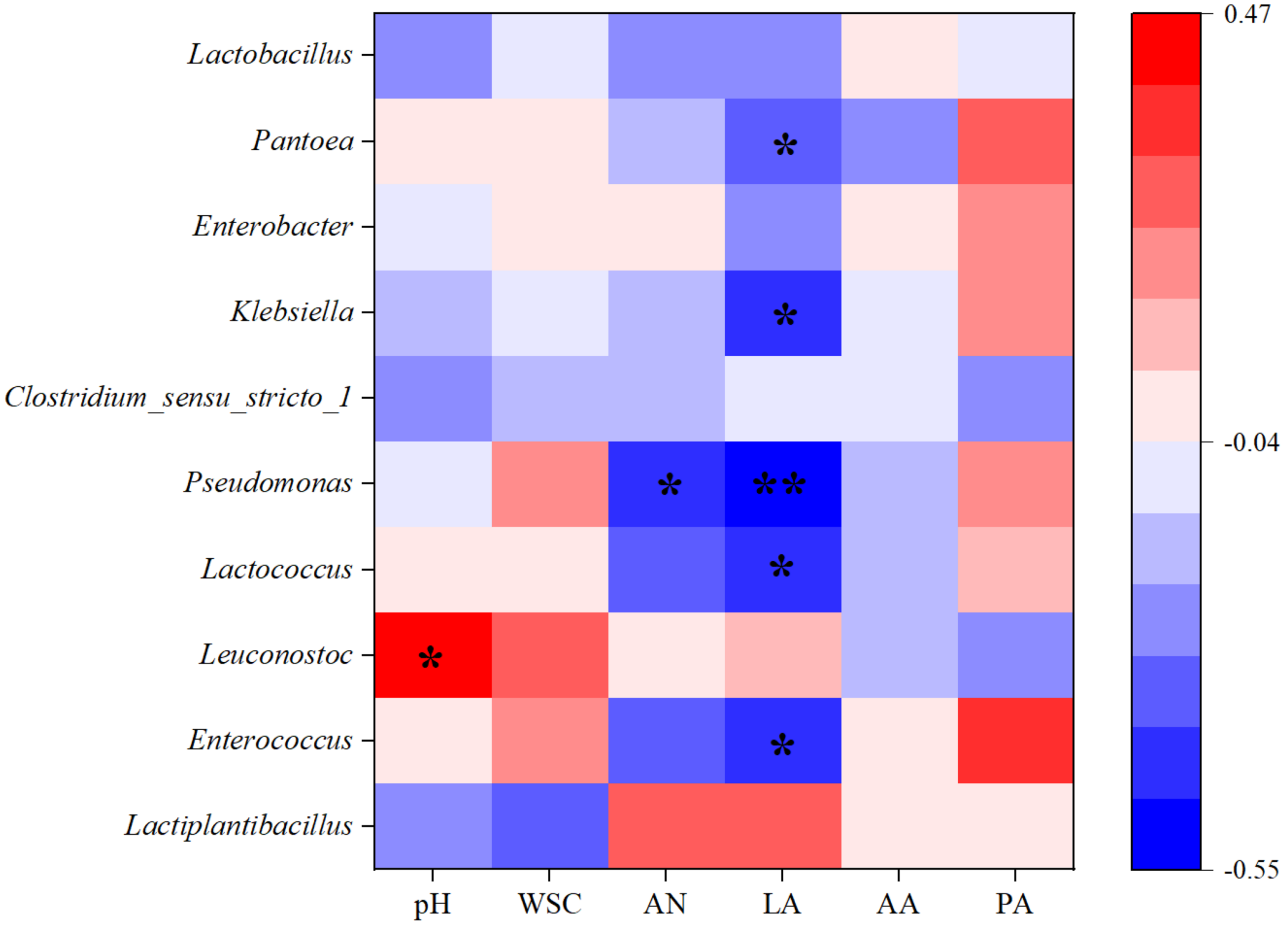
Heatmap of Spearman correlations between bacterial abundances and silage fermentation parameters on day 60. WSC, water-soluble carbohydrate; AN, ammonia nitrogen; LA, lactic acid; AA, acetic acid; PA, propionic acid. The colours in the heatmaps indicate the Spearman correlation coefficient r, which ranges from −0.55 to 0.47. r < 0 indicates a negative correlation, and r > 0 indicates a positive correlation. ‘^*^’ and ‘^**^’ represent *p* < 0.05 and *p* < 0.01, respectively.

The Spearman correlation between silage parameters and bacterial communities was established to elucidate their associations, which were then displayed in the form of a heatmap. Figure 5 shows the correlation between metabolites and microbial diversity. Previous studies have shown that the metabolites produced during silage fermentation could affect the bacterial community, and the metabolites can also be improved through microbial diversity, thus affecting the quality of silage (Li et al., 2022). In addition, previous studies have shown that some metabolites are generally positively associated with favourable microorganisms and negatively associated with undesirable bacteria during silage fermentation (Ren et al., 2019). In this study, the dominant community *Lactiplantibacillus* was positively correlated with the concentration of LA, indicating that it could increase the production of LA, but it was negatively correlated with pH. Therefore, the higher relative abundance of *Lactiplantibacillus* in 0.2E and 0.8E resulted in their lower pH compared with that of CK (Table 2; Figure 2). However, the relative abundance of *Pseudomonas* was negatively correlated with the LA concentration (*p* < 0.01), and those of *Enterococcus, Lactococcus*, *Klebsiella*, and *Pantoea* were negatively correlated with the LA concentration (*p* < 0.05), which is similar to the results of Mu et al. (2020).

## Conclusion

Three additives had different effects on the fermentation quality and bacterial community of paper mulberry silage. As a protease inhibitor, adding EDTA reduced protein decomposition and preserved more WSCs, moreover, it inhibited the activity of undesirable microorganisms, resulting in a decrease in the relative abundance of *Enterococcus* (especially in 0.2E group). With the increase of EDTA concentration, the fermentation process of paper mulberry silage became slower. In the early stage, propionic acid changed the silage microbiota, cause the pH to decrease rapidly. Adding PA increased the abundance of beneficial microorganisms such as *Lactiplantibacillus*, and the addition of low PA concentration led to a higher LA concentration and a lower PA concentration. The pH of silage treated with a high concentration of CA decreased rapidly in the early stage, more WSC concentration was also preserved. However, for the safe and clean production of paper mulberry silage, further investigation on the changes in its bioactive components when treated with different organic acids is needed.

## Data availability statement

The datasets presented in this study can be found in online repositories. The names of the repository/repositories and accession number(s) can be found at: BioProject, accession number PRJNA881337.

## Author contributions

ML, QY, and JX designed the study, wrote the manuscript and performed the experiments. HS, QC, YX, and CW conducted the statistical analysis. PL, CC, and YZ were involved in the revision of the manuscript. All authors contributed to the article and approved the submitted version.

## Funding

This work was supported by Guizhou provincial Science and Technology Projects [grant number: QKHJC-ZK(2022)General 156] and the National Key Research and Development Program of China [grant number: 2021YFD1300300].

## Conflict of interest

The authors declare that the research was conducted in the absence of any commercial or financial relationships that could be construed as a potential conflict of interest.

## Publisher’s note

All claims expressed in this article are solely those of the authors and do not necessarily represent those of their affiliated organizations, or those of the publisher, the editors and the reviewers. Any product that may be evaluated in this article, or claim that may be made by its manufacturer, is not guaranteed or endorsed by the publisher.

## Footnotes

^1^ https://magic.novogene.com/customer/main
